# Plasma acylcarnitine in elderly Taiwanese: as biomarkers of possible sarcopenia and sarcopenia

**DOI:** 10.1186/s12877-023-04485-x

**Published:** 2023-11-22

**Authors:** Chi-Jen Lo, Chih-Ming Lin, Chun-Ming Fan, Hsiang-Yu Tang, Han-Fang Liu, Hung-Yao Ho, Mei-Ling Cheng

**Affiliations:** 1 Metabolomics Core Laboratory, Healthy Aging Research Center, Chang Gung University, Taoyuan City, 33302 Taiwan; 2 Division of Internal Medicine, Chang Gung Memorial Hospital, Taipei, 105 Taiwan; 3 Department of Health Management, Chang Gung Health and Culture Village, Taoyuan City, 333 Taiwan; 4 Clinical Metabolomics Core Laboratory, Chang Gung Memorial Hospital, Taoyuan City, 33302 Taiwan; 5 Graduate Institute of Biomedical Sciences, College of Medicine, Chang Gung University, Taoyuan City, 33302 Taiwan; 6 Department of Biomedical Sciences, College of Medicine, Chang Gung University, Taoyuan City, 33302 Taiwan

**Keywords:** Sarcopenia, Metabolism, Muscle mass, Carnitine

## Abstract

**Background:**

Sarcopenia is defined as the disease of muscle loss and dysfunction. The prevalence of sarcopenia is strongly age-dependent. It could bring about disability, hospitalization, and mortality. The purpose of this study was to identify plasma metabolites associated with possible sarcopenia and muscle function to improve disease monitoring and understand the mechanism of muscle strength and function decline.

**Methods:**

The participants were a group of healthy older adult who live in retirement homes in Asia (Taiwan) and can manage their daily lives without assistance. The participants were enrolled and divided into four groups: control (Con, n = 57); low physical function (LPF, n = 104); sarcopenia (S, n = 63); and severe sarcopenia (SS, n = 65) according to Asian countries that used Asian Working Group for Sarcopenia (AWGS) criteria. The plasma metabolites were used and the results were calculated as the difference between the control and other groups.

**Results:**

Clinical parameters, age, gender, body mass index (BMI), hand grip strength (HGS), gait speed (GS), blood urea nitrogen (BUN), hemoglobin, and hematocrit were significantly different between the control and LPF groups. Metabolite patterns of LPF, S, and SS were explored in our study. Plasma kynurenine (KYN) and acylcarnitines (C0, C4, C6, and C18:1-OH) were identified with higher concentrations in older Taiwanese adults with possible sarcopenia and S compared to the Con group. After multivariable adjustment, the data indicate that age, BMI, and butyrylcarnitine (C4) are more important factors to identify individuals with low physical function and sarcopenia.

**Conclusion:**

This metabolomic study raises the importance of acylcarnitines on muscle mass and function. It suggests that age, BMI, BUN, KYN, and C4/Cr can be important evaluation markers for LPF (AUC: 0.766), S (AUC: 0.787), and SS (AUC: 0.919).

**Supplementary Information:**

The online version contains supplementary material available at 10.1186/s12877-023-04485-x.

## Background

Sarcopenia is defined by muscle loss and dysfunction, and classified, based on the cause, into primary and secondary types. Primary sarcopenia is mainly age-related, where both loss of muscle mass and function occurs in older individuals [1–3], while secondary sarcopenia accompanies an underlying disease [4, 5]. Sarcopenia causes concern because it may result in many adverse outcomes such as disability, hospitalization, mortality, and poor quality of life [6–9]. Since the prevalence of sarcopenia significantly increases with age [10–12], it is important to aged or hyper-aged society country all around the world Early identification of people on the way to or at risk of sarcopenia, enables earlier lifestyle interventions and health promotion [13]. In addition, “possible sarcopenia,” which defined by Asian Working Group for Sarcopenia (AWGS) refers to poor muscle strength or low physical performance [14]. It highlights the importance of early detection and intervention in this subclinical group for the prevention of sarcopenia.

Metabolomics is the technology that enables comprehensive analyses of metabolites in a biological system [15, 16]. Because metabolites are expressed downstream of gene expression and reveal disease phenotypes, the metabolomics approach may improve diagnosis and prognosis, particularly in complex degenerative diseases in human [17]. Several metabolomics studies suggest that certain metabolic profiles, such as those of amino acids, acylcarnitines, lipids, and gut microbiome-related metabolites are related to muscle mass [18–20] and physical function [21–26]. Although skeletal muscle strength and mass are both still considered fundamental to a definitive clinical diagnosis, the age-related decline in muscle mass does not occur in parallel with a decline in muscle function [27]. In the previous study, inflammatory indexes (homocysteine and high-sensitive C-reactive protein) were positive correlation with sarcopenia in Chinese older adults with type 2 diabetes mellitus [28]. Myostatin might be a biomarker of sarcopenia undergoing rehabilitation and amino acid supplementation [29]. Therefore, we analyzed muscle mass and muscle function in combination with plasma metabolites to identify specific traits of circulating metabolites in relatively healthy and older Taiwanese. The participants of this study who displayed normal muscle mass but low physical function (LPF) were divided from possible sarcopenia to further investigate the progression of sarcopenia. The main aim of this study is to identify the metabolite profile which can distinguish normal controls from LPF, sarcopenia, and severe sarcopenia in older Asian.

The metabolite of amino acid such as kynurenine (KYN) was increases during age [30] and circulating KYN may serve as a biomarker related to the risk of frailty [31]. Plasma acylcarnitines which associated to lipid metabolism increase with aging [32], are associated with physical performance in older adults [33], and can be biomarkers of sarcopenia in preoperative gastrointestinal cancer patients [34]. The association of increased plasma concentrations of medium- and long-chain acylcarnitines and lower-extremity functional impairment (LEFI) under multiple organ function measurement was revealed [35]. Both circulation KYN and acylcarnitines may serve as biomarkers in age-related diseases such as frailty [36, 37]. In this study, the data after multivariable adjustment indicate that age, BMI and butyrylcarnitine (C4) are more important factors to identify individuals with low physical function or sarcopenia from the early to severe stages. These metabolic signatures, not only provide important knowledge to better understand the pathogenesis of sarcopenia, but can also be applied to monitor the disease status as well as evaluate the beneficial effects of intervention. Blood biomarkers of possible sarcopenia or sarcopenia are not yet available in clinical practice. Early intervention might reduce the incidence of sarcopenia and promote health in older adults. Consequently, the key biomarkers identified for the early stage of sarcopenia are an important preventive strategy.

## Methods

### Study population and design

The study protocol was approved by the Institutional Review Board of [blinded for review] Hospital. Written informed consent was obtained from all participants. Blood samples were collected during the participants’ annual physical and health examinations (02/20/2014 ~ 11/20/2014). The study enrolled a total of 491 subjects, aged 65 years or older, who did not require nursing assistance and lived in a retirement home located in northern Taiwan. There are 289 clinical data and metabolites concentration could be analyzed, and the exclusion criteria were lacking of clinical biochemical data (n = 186) and even suffered from cancers (n = 16). Plasma samples were obtained from participants for hematological, biochemical, and metabolomics studies. Handgrip strength (HGS), gait speed (GS), and muscle mass were also measured to identify the risk factors for sarcopenia. Study flow diagram was provided in Figure S1. The total of 289 subjects could be separated to four groups: control group (Con, n = 57, who had normal hand grip strength: men ≥ 28 kg, women ≥ 18 kg, and normal gait speed ≥ 1.0 m/s), low physical function (LPF, n = 104, possible sarcopenia with normal muscle mass: men ≥ 7.0 kg/m^2^, women ≥ 5.4 kg/m^2^), sarcopenia (S, n = 63), and severe sarcopenia (SS, n = 65, low HGS and GS).

### Assessment of Sarcopenia and muscle mass and function determination

In consideration of the anthropometric differences between Asian and European, we defined four stage of sarcopenia according to the diagnostic algorithm of Asian Working Group for Sarcopenia (AWGS), which resembles European Working Group on Sarcopenia in Older People (EWGSOP). Based on AWGS 2019 criteria, low physical function (LPF) was defined as the presence of low muscle strength or low physical performance; sarcopenia (S) was defined as the presence of low muscle mass, plus low muscle strength or low physical performance; severe sarcopenia (SS) was defined as the presence of low muscle mass, low muscle strength and low physical performance [14].

The muscle mass of each participant was measured using dual energy X-ray absorptiometry (GE Lunar iDXATM; GE Healthcare, Madison, WI, USA), and the appendicular skeletal muscle mass index (ASMI) from dual energy X-ray absorptiometry was calculated in units of kilogram per meter squared. Low muscle mass was defined as ASMI less than 7.0 kg/m^2^ for male and less than 5.4 kg/m^2^ for female [14].

Muscle strength was assessed by hand grip strength (HGS) using a hand dynamometer (Jamar Plus + Digital Hand Dynamometer; Sammons Preston, Bollingbrook, IL, USA). Two trials for each hand were performed and the highest reading was used in the analyses. Low muscle strength was defined as grip strength < 28 kg or < 18 kg for male and female, respectively [14].

Physical performance was assessed by gait speed (GS). Each participant was asked to walk a distance of 4 m in order to measure his or her GS. Two trials were performed and the shortest walking time was used in the analyses. Low physical performance was defined as a usual GS < 1.0 m/s for both male and female [14].

### Determining the plasma metabolite profile using ultra-high performance liquid chromatography-tandem mass

Metabolites in plasma samples were analyzed using a commercially available kit (BIOCRATES Life Sciences AG, Austria). Briefly, each 10 µL of plasma was prepared and processed according to the manufacturer’s instructions as previously described [38]. Biogenic amines were measured by ultra-high performance liquid chromatography-tandem mass spectrometry and lipid species were quantified by flow injection analysis coupled with tandem mass spectrometry. Chromatographic separation was performed on an Acquity BEH C18 column (75 × 2.1 mm; particle size, 1.7 μm) (Waters crop., Milford, CT, USA) at 50 °C with a flow rate of 0.9 mL/min. Mobile phase A comprised a mixture of water with 0.2% formic acid, while mobile phase B comprised acetonitrile with 0.2% formic acid. The linear gradient was set as follows: 0–0.38 min, 0% B; 0.38–3 min, 0–15% B; 3–5.4 min, 15–70% B; 5.4–5.93 min, 100% B; and 5.93–6.6 min, 0% B for re-equilibration. The parameters of mass were as follows: capillary voltage 3.2 kV; desolvation gas flow 1200 L/h; desolvation temperature 650 °C; source temperature 150 °C; and voltage 10 V. For flow injection analysis, a low of 0.03 mL/min was used with commercial solvent and processed as previously described [39]. Concentrations of metabolite were calculated and expressed in µM.

### Statistical analysis

The baseline characteristics and metabolite concentrations are presented as mean ± standard deviation for continuous variables and as counts (percentages) for categorical variables. Comparisons between the control group and other groups (i.e. LPF, S, and SS) were carried out using an independent student’s *t*-test. Multivariate logistic regression was used to analyze the difference between the control group and other groups in each baseline characteristic and metabolite concentrations when adjusting for age, sex and comorbidities, including hypertension, diabetes, hyperlipidemia, coronary artery disease (CAD), cancer, stroke, chronic kidney disease (CKD), chronic obstructive pulmonary disease (COPD), and osteoporosis. Odds ratios (ORs) and 95% confidence interval (CI) were calculated. All statistical analyses were two-sided and were performed using SPSS software version 19.0 (SPSS, Chicago, IL, USA). A *P* value of < 0.05 was considered statistically significant. The receiver operating characteristic (ROC) curves were calculated by linear SVM (support vector machine) classification method. The AUC (area under curve) value was selected as the model for LPF, S, and SS prediction. The AUC was calculated using MetaboAnalyst (www.metaboanalyst.ca).

## Results

### Baseline characteristics

The average age of participants was 81.85 years (females, n = 166 [57.4%]; males, n = 123 [42.6%]). The 289 subjects were eligible to participate. Then, targeted metabolomics analysis was performed for 289 samples with completed measurement data. According to Asian Working Group for Sarcopenia (AWGS) criteria [14], the study participants were divided into four groups: Control (Con, participants who had normal hand grip strength: men ≥ 28 kg, women ≥ 18 kg, and normal gait speed ≥ 1.0 m/s, n = 57), low physical function (LPF, possible sarcopenia with normal muscle mass: men ≥ 7.0 kg/m^2^, women ≥ 5.4 kg/m^2^, n = 104), sarcopenia (S, n = 63), and severe sarcopenia (SS, low HGS and GS, n = 65) based on the muscle mass, muscle strength, and physical performance (Figure S1). The baseline characteristics and laboratory data with significant differences were noted from the Con group to participants in the LPF, S, or SS groups (Table 1). Compared with control subjects, participants with SS had remarkably higher concentrations of BUN (21.9 ± 11.3 mg/dL, P value < 0.0001), creatinine (1.1 ± 0.7 mg/dL, P value = 0.0069), uric acid (6.1 ± 1.6 mg/dL, P value = 0.0155), and cortisol (16.1 ± 4.8 ug/dL, P value = 0.0053). Conversely, SS subjects had lower concentrations of hemoglobin (12.5 ± 1.4 g/dL, P value < 0.0001), and red blood cells (RBCs) (4.2 ± 0.5 10^6^/ul, P value = 0.0092), and hematocrit (RBC, %) (37.4 ± 4.0%, P value, 0.0001). The proportions of chronic kidney disease (CKD) (17%), chronic obstructive pulmonary disease (COPD) (34%), and osteoporosis (45%) were higher in the SS group than control group. In terms of age, they were older than the control subjects (Con, 75.9 ± 7.5 year.; LPF, 81.9 ± 6.8 year.; S, 82.5 ± 5.8 year.; SS, 86.3 ± 4.2 year.). The percentage of males was lower in the LPF group (24%).

**Table 1:** Characteristics of study population according to sarcopenia

	Con	LPF	S	SS	*P* value		
Parameter	n = 57	n = 104	n = 63	n = 65	LPF vs. Con	S vs. Con	SS vs. Con
Age	75.9 ± 7.5	81.9 ± 6.8	82.5 ± 5.8	86.3 ± 4.2	**0.0000**	**0.0000**	**0.0000**
Male (%)	46%	24%	51%	62%	**0.0118**	0.5707	0.0782
height (cm)	161.2 ± 8.2	154.6 ± 6.6	158.2 ± 8.2	158.7 ± 8.9	**0.0000**	0.0471	0.1126
weight (kg)	61.1 ± 11.6	60.3 ± 8.8	55.2 ± 10.2	54.5 ± 10.5	0.6437	**0.0038**	**0.0013**
BMI (kg/m^2^)	23.4 ± 3.2	25.2 ± 3.1	21.8 ± 2.8	21.5 ± 3.0	**0.0008**	**0.0040**	**0.0012**
ASMI (kg/m^2^)	6.4 ± 0.9	6.4 ± 0.7	5.7 ± 0.8	5.6 ± 0.8	0.9968	**0.0000**	**0.0000**
HGS (kg)	26.3 ± 6.3	15.4 ± 5.5	18.2 ± 6.7	16.4 ± 5.7	**0.0000**	**0.0000**	**0.0000**
GS(m/sec)	1.3 ± 0.3	0.9 ± 0.3	1.2 ± 0.2	0.7 ± 0.2	**0.0000**	**0.0062**	**0.0000**
glucose (mg/dL)	97.4 ± 20.2	99.1 ± 15.4	97.7 ± 20.7	103.6 ± 36.5	0.5892	0.9347	0.2445
HbA1c (%)	5.8 ± 0.5	5.9 ± 0.6	5.8 ± 0.6	6.0 ± 1.0	0.4072	0.9269	0.2863
LDL (mg/dL)	111.5 ± 34.4	106.3 ± 31.1	106.7 ± 28.1	99.5 ± 25.4	0.3383	0.4021	**0.0330**
HDL (mg/dL)	53.7 ± 12.0	53.2 ± 12.2	54.9 ± 14.1	52.8 ± 13.1	0.8077	0.6377	0.6792
Chol (mg/dL)	185.0 ± 38.7	182.7 ± 37.3	180.0 ± 34.6	172.5 ± 31.7	0.7108	0.4517	0.0517
TG(mg/dL)	102.5 ± 58.7	109.1 ± 54.1	86.9 ± 34.3	95.8 ± 60.9	0.4723	0.0838	0.5394
Albumin (g/dL)	4.4 ± 0.3	4.3 ± 0.3	4.3 ± 0.2	4.3 ± 0.2	0.6934	0.5767	0.0565
T-protein (g/dL)	7.0 ± 0.4	6.9 ± 0.4	7.0 ± 0.4	7.0 ± 0.4	0.5447	0.8991	0.6439
T-bilirubin (mg/dL)	0.7 ± 0.3	0.7 ± 0.3	0.8 ± 0.4	0.8 ± 0.4	0.8765	0.4802	0.5600
BUN (mg/dL)	15.2 ± 3.9	17.8 ± 9.1	17.2 ± 6.4	21.9 ± 11.3	**0.0097**	**0.0355**	**0.0000**
Creatinine (mg/dL)	0.8 ± 0.2	0.9 ± 0.9	0.9 ± 0.5	1.1 ± 0.7	0.3428	0.0769	**0.0069**
Uric acid (mg/dL)	5.4 ± 1.3	5.7 ± 1.6	5.7 ± 1.6	6.1 ± 1.6	0.2446	0.3592	**0.0155**
AST/GOT (U/L)	29.7 ± 19.4	26.7 ± 9.7	28.7 ± 10.0	30.6 ± 40.3	0.2818	0.7187	0.8776
ALT/GPT (U/L)	23.5 ± 21.8	19.7 ± 16.4	19.9 ± 12.9	23.7 ± 55.6	0.2593	0.2798	0.9786
ALKP (U/L)	67.7 ± 24.2	68.1 ± 19.2	61.7 ± 16.6	65.5 ± 22.2	0.9255	0.1159	0.5969
TSH (uIU/ml)	2.4 ± 1.7	2.6 ± 2.3	2.4 ± 1.9	2.8 ± 2.2	0.6411	0.9895	0.3386
T4 (ug/dL)	8.6 ± 1.5	8.0 ± 1.7	8.2 ± 1.6	8.2 ± 1.7	0.0303	0.1056	0.1085
Cortisol (ug/dL)	13.6 ± 4.9	13.8 ± 4.7	13.5 ± 4.6	16.1 ± 4.8	0.7915	0.9336	**0.0053**
Vit B12 (pg/ml)	754.1 ± 538.5	735.3 ± 503.6	694.5 ± 375.3	890.6 ± 702.3	0.8270	0.4904	0.2294
WBC (10^3^/ul)	5.3 ± 1.5	5.4 ± 1.5	5.5 ± 1.4	5.7 ± 1.4	0.7553	0.4920	0.2367
RBC (10^6^/ul)	4.4 ± 0.4	4.3 ± 0.5	4.3 ± 0.4	4.2 ± 0.5	0.1865	0.1047	**0.0092**
Hemoglobin g/dl	13.5 ± 1.2	13.0 ± 1.6	13.1 ± 1.6	12.5 ± 1.4	**0.0144**	0.1067	**0.0000**
RBC (%)	40.4 ± 3.3	39.0 ± 4.2	39.3 ± 4.5	37.4 ± 4.0	**0.0367**	0.1499	**0.0000**
Platelets (10^3^/ul)	200.5 ± 47.3	205.5 ± 56.2	196.3 ± 57.0	193.7 ± 45.3	0.5632	0.6641	0.4206
Na (mEq/L)	142.1 ± 2.8	142.3 ± 2.6	141.8 ± 2.4	141.1 ± 3.3	0.5636	0.6344	0.0790
K (mEq/L)	4.3 ± 0.4	4.3 ± 0.4	4.3 ± 0.5	4.3 ± 0.4	0.5925	0.5723	0.5034
Cl (mEq/L)	105.8 ± 3.1	106.1 ± 2.9	105.8 ± 2.9	104.6 ± 4.0	0.5795	0.9289	0.0659
Ca (mg/dL)	9.1 ± 0.4	9.2 ± 0.4	9.1 ± 0.3	9.1 ± 0.4	0.6111	0.8089	0.4931
Hypertension (%)	40%	66%	53%	57%	**0.0014**	0.1598	0.0677
Diabetes (%)	16%	28%	26%	25%	0.0839	0.1802	0.2282
Hyperlipidemia (%)	30%	38%	35%	28%	0.3281	0.5112	0.7950
CAD (%)	5%	9%	6%	14%	0.4334	0.7831	0.1122
Stroke (%)	4%	10%	5%	9%	0.1583	0.7179	0.2027
CKD (%)	2%	13%	15%	17%	**0.0207**	**0.0122**	**0.0050**
COPD (%)	18%	15%	26%	34%	0.7218	0.2759	**0.0411**
Osteoporosis (%)	19%	29%	34%	45%	0.1836	0.0733	**0.0030**

Con: control; LPF: loss of physical function; S: sarcopenia; SS: severe sarcopenia. Group definition is according to the Asian Working Group for Sarcopenia (AWGS) 2019 definition and algorithm

BMI: body mass index; ASMI: appendicular skeletal muscle mass index; HGS: handgrip strength; GS: gait speed; HbA1c: glycosylated hemoglobin; LDL: low density lipoprotein; HDL: high density lipoprotein; Chol: cholesterol; TG: triglyceride; BUN: blood urea nitrogen; AST: aspartate aminotransferase; ALT: alanine aminotransferase; ALKP: alkaline phosphatase; WBC: white blood cell; RBC: red blood cell; T4: 3,5,3’,5’-L-tetraiodothyronine; TSH: thyrotropin; CAD: coronary artery disease; CKD: chronic kidney disease; COPD: chronic obstructive pulmonary disease

**Fig. 1:**
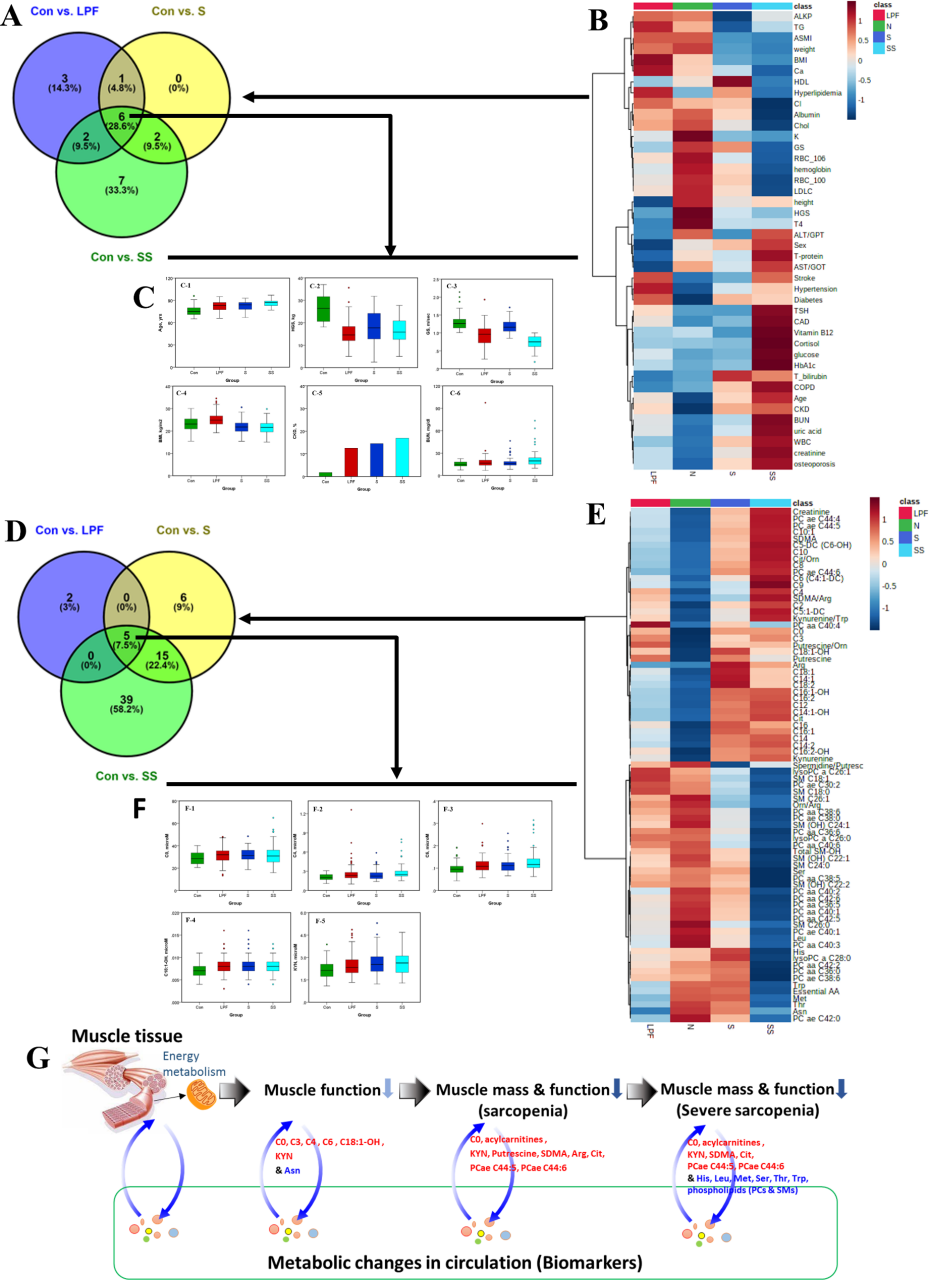
Clinical and metabolic profiles in low physical function (LPF), sarcopenia (S), and severe sarcopenia (SS) and control (Con) group. The selected clinical parameters that were markedly different in LPF, S and SS when compared with Con group. These parameters were presented using a Venn diagram (**A**) and heatmap (**B**). (**C**) The six significant markers including age (C-1), hand grip strength (HGS) (C-2), gait speed (GS) (C-3), BMI (C-4), CKD (C-5), and BUN (C-6) were presented in each group (Con, LPF, S, and SS). Plasma samples from participants of different group were collected to determine metabolite concentrations by UPLC-MSMS. A Venn diagram presented the metabolites that were significantly different in each group (LPF, S and SS) when compared with control group (**D**). The heatmap revealed high variability of metabolites in each group (**E**). (**F**) The overlap in significant metabolites including C0 (F-1), C4 (F-2), C6 (F-3), C18:1-OH (F-4) and KYN (F-5) were presented by box plots for plasma levels in each group (Con, LPF, S, and SS). (**G**) The summarized scheme of changed metabolites in each group was shown. In Venn diagram, Con vs. LPF, labeled by blue violet color; Con vs. S, labeled by yellow color; Con vs. SS, labeled by green color. In heatmap and bar plot, Con, LPF, S, and SS were labeled by green, red, blue, and sky blue, respectively. All the selected clinical parameters and metabolites are p < 0.05 between the control group and each of the LPF, S, and SS group

We examined the clinical parameters in all participants. To test whether differences exist, we compared clinical parameters in the LPF, S, and SS groups with the Con group. All the significant clinical parameters between control group compared with each group (LPF, S, or SS) were showed by Venn diagram (Fig. 1A) and heatmap (Fig. 1B). There are 12, 9, and 17 differential clinical parameters found in LPF, S, and SS groups when compared with the Con group (*P* value < 0.05), respectively (Table 1; Fig. 1). The heatmap revealed high variability in each group (Fig. 1B). In the Venn diagram, the six significant markers including age (C-1), hand grip strength (HGS) (C-2), gait speed (GS) (C-3), BMI (C-4), CKD (C-5), and BUN (C-6) were presented in each group (LPF, S, or SS) relative to the control group (Fig. 1C). These clinical parameters (age and BMI) which were listed in Table 2 for further multivariable analysis were important risk factor for each group (LPF, S, or SS) relative to the Con group.

**Table 2:** Concentration of metabolites significantly differentially expressed among control, low physical function, sarcopenia, and severe sarcopenia groups

	Con	LPF	S	SS	*P* value		
Parameter	n = 57	n = 104	n = 63	n = 65	LPF vs. Con	S vs. Con	SS vs. Con
C0	28.995 ± 5.357	31.476 ± 7.090	31.657 ± 5.791	31.651 ± 9.125	**0.0136**	**0.0103**	**0.0494**
C2	7.580 ± 2.438	8.265 ± 2.693	8.258 ± 2.425	8.583 ± 2.562	0.1129	0.1301	**0.0293**
C3	0.305 ± 0.091	0.343 ± 0.117	0.335 ± 0.097	0.343 ± 0.128	**0.0228**	0.0797	0.0597
C4	0.207 ± 0.046	0.264 ± 0.136	0.244 ± 0.086	0.286 ± 0.123	**0.0002**	**0.0038**	**< 0.0000**
C6 (C4:1-DC)	0.098 ± 0.029	0.113 ± 0.036	0.116 ± 0.036	0.133 ± 0.054	**0.0056**	**0.0027**	**< 0.0000**
C5:1-DC	0.012 ± 0.004	0.014 ± 0.005	0.014 ± 0.006	0.015 ± 0.007	0.0563	0.1325	**0.0115**
C5-DC (C6-OH)	0.032 ± 0.007	0.034 ± 0.014	0.035 ± 0.012	0.037 ± 0.014	0.3352	0.1000	**0.0144**
C8	0.254 ± 0.078	0.270 ± 0.088	0.293 ± 0.102	0.309 ± 0.117	0.2574	**0.0213**	**0.0029**
C9	0.028 ± 0.009	0.029 ± 0.014	0.029 ± 0.010	0.032 ± 0.013	0.3846	0.4016	**0.0313**
C10	0.316 ± 0.116	0.339 ± 0.137	0.367 ± 0.125	0.394 ± 0.171	0.2904	**0.0234**	**0.0033**
C10:1	0.461 ± 0.086	0.490 ± 0.121	0.514 ± 0.133	0.538 ± 0.132	0.0748	**0.0099**	**0.0002**
C12	0.111 ± 0.031	0.117 ± 0.036	0.125 ± 0.030	0.127 ± 0.040	0.3007	**0.0175**	**0.0198**
C14	0.034 ± 0.006	0.036 ± 0.006	0.037 ± 0.007	0.037 ± 0.007	0.1071	**0.0135**	**0.0100**
C14:1	0.075 ± 0.014	0.078 ± 0.018	0.082 ± 0.016	0.079 ± 0.017	0.2606	**0.0147**	0.1369
C14:1-OH	0.016 ± 0.004	0.016 ± 0.005	0.017 ± 0.004	0.018 ± 0.004	0.3896	**0.0383**	**0.0204**
C14:2	0.052 ± 0.017	0.057 ± 0.024	0.061 ± 0.021	0.062 ± 0.024	0.1384	**0.0097**	**0.0073**
C16	0.122 ± 0.025	0.130 ± 0.029	0.134 ± 0.025	0.132 ± 0.032	0.0941	**0.0097**	0.0549
C16:1	0.031 ± 0.008	0.033 ± 0.011	0.035 ± 0.009	0.035 ± 0.010	0.0754	**0.0033**	**0.0084**
C16:1-OH	0.012 ± 0.002	0.012 ± 0.002	0.013 ± 0.002	0.013 ± 0.002	0.3839	**0.0423**	**0.0369**
C16:2	0.015 ± 0.004	0.016 ± 0.004	0.017 ± 0.005	0.017 ± 0.005	0.2288	**0.0086**	**0.0067**
C16:2-OH	0.013 ± 0.002	0.014 ± 0.002	0.014 ± 0.002	0.014 ± 0.002	0.0585	**0.0241**	**0.0099**
C18:1	0.149 ± 0.032	0.155 ± 0.039	0.161 ± 0.033	0.157 ± 0.043	0.3185	**0.0404**	0.2516
C18:1-OH	0.007 ± 0.001	0.008 ± 0.002	0.008 ± 0.002	0.008 ± 0.002	**0.0183**	**0.0074**	**0.0417**
C18:2	0.104 ± 0.022	0.108 ± 0.027	0.116 ± 0.030	0.111 ± 0.031	0.2856	**0.0128**	0.1297
Arginine	72.972 ± 19.257	73.962 ± 19.533	83.427 ± 22.597	80.025 ± 20.450	0.7578	**0.0077**	0.0532
Asparagine	50.747 ± 8.329	47.488 ± 9.389	50.179 ± 7.724	47.920 ± 8.160	**0.0300**	0.6990	0.0610
Citrulline	36.928 ± 10.587	39.452 ± 17.063	43.506 ± 11.719	44.506 ± 15.105	0.2495	**0.0017**	**0.0016**
Histidine	96.351 ± 10.459	96.015 ± 14.061	98.137 ± 12.911	91.843 ± 12.517	0.8640	0.4099	**0.0343**
Leucine	139.100 ± 26.761	133.380 ± 26.365	134.317 ± 21.967	128.675 ± 29.997	0.1922	0.2850	**0.0463**
Methionine	27.739 ± 5.885	26.265 ± 5.191	27.713 ± 5.536	25.677 ± 5.280	0.1026	0.9802	**0.0436**
Serine	125.725 ± 18.161	124.340 ± 23.136	125.089 ± 23.005	117.474 ± 26.469	0.6761	0.8678	**0.0450**
Threonine	128.232 ± 24.331	120.685 ± 24.950	126.475 ± 20.241	116.806 ± 24.930	0.0660	0.6669	**0.0119**
Tryptophan	59.411 ± 11.585	56.603 ± 12.263	59.479 ± 10.691	54.400 ± 10.940	0.1586	0.9731	**0.0155**
Kynurenine	2.196 ± 0.614	2.493 ± 0.720	2.622 ± 0.832	2.677 ± 0.817	**0.0092**	**0.0017**	**0.0003**
Putrescine	0.141 ± 0.071	0.174 ± 0.327	0.172 ± 0.081	0.162 ± 0.081	0.3358	**0.0279**	0.1316
SDMA	0.707 ± 0.169	0.795 ± 0.445	0.855 ± 0.351	0.933 ± 0.414	0.0760	**0.0036**	**0.0001**
lysoPC a C26:0	0.176 ± 0.038	0.175 ± 0.043	0.170 ± 0.032	0.163 ± 0.029	0.9655	0.4097	**0.0468**
lysoPC a C26:1	0.109 ± 0.030	0.112 ± 0.033	0.105 ± 0.024	0.099 ± 0.028	0.5669	0.4482	**0.0478**
lysoPC a C28:0	0.267 ± 0.048	0.264 ± 0.066	0.275 ± 0.065	0.248 ± 0.054	0.7170	0.4601	**0.0449**
PC aa C36:0	4.581 ± 1.405	4.499 ± 1.410	4.618 ± 2.142	4.055 ± 1.411	0.7230	0.9102	**0.0417**
PC aa C36:5	22.635 ± 15.857	19.799 ± 12.469	20.564 ± 16.167	15.954 ± 7.978	0.2462	0.4808	**0.0051**
PC aa C36:6	0.701 ± 0.355	0.684 ± 0.320	0.637 ± 0.329	0.525 ± 0.200	0.7617	0.3121	**0.0014**
PC aa C38:5	40.782 ± 12.515	40.209 ± 10.978	40.022 ± 11.733	36.232 ± 9.079	0.7633	0.7319	**0.0253**
PC aa C38:6	90.418 ± 27.590	86.529 ± 23.444	83.000 ± 25.168	73.837 ± 21.279	0.3463	0.1262	**0.0004**
PC aa C40:1	0.546 ± 0.216	0.512 ± 0.194	0.520 ± 0.216	0.471 ± 0.159	0.3023	0.5141	**0.0326**
PC aa C40:2	0.482 ± 0.358	0.422 ± 0.321	0.453 ± 0.287	0.365 ± 0.176	0.2761	0.6188	**0.0273**
PC aa C40:3	0.776 ± 0.450	0.687 ± 0.360	0.703 ± 0.345	0.619 ± 0.274	0.1691	0.3225	**0.0246**
PC aa C40:4	2.322 ± 0.572	2.543 ± 0.634	2.447 ± 0.636	2.379 ± 0.654	**0.0307**	0.2627	0.6132
PC aa C40:6	32.863 ± 10.153	32.138 ± 9.371	29.775 ± 8.473	26.690 ± 8.902	0.6493	0.0720	**0.0005**
PC aa C42:2	0.337 ± 0.124	0.326 ± 0.123	0.340 ± 0.145	0.292 ± 0.089	0.6190	0.8796	**0.0265**
PC aa C42:5	0.432 ± 0.240	0.381 ± 0.193	0.392 ± 0.252	0.315 ± 0.123	0.1473	0.3802	**0.0013**
PC aa C42:6	0.466 ± 0.197	0.433 ± 0.158	0.443 ± 0.228	0.380 ± 0.103	0.2452	0.5524	**0.0040**
PC ae C30:2	0.066 ± 0.014	0.067 ± 0.016	0.064 ± 0.014	0.061 ± 0.014	0.7735	0.3430	**0.0355**
PC ae C38:0	1.826 ± 0.767	1.700 ± 0.690	1.639 ± 0.600	1.426 ± 0.489	0.2861	0.1375	**0.0010**
PC ae C38:6	6.671 ± 1.688	6.614 ± 2.004	6.774 ± 2.496	5.970 ± 1.820	0.8536	0.7903	**0.0300**
PC ae C40:1	1.054 ± 0.307	0.998 ± 0.287	1.007 ± 0.255	0.939 ± 0.308	0.2531	0.3625	**0.0411**
PC ae C42:0	0.834 ± 0.206	0.777 ± 0.164	0.806 ± 0.168	0.756 ± 0.129	0.0736	0.4103	**0.0154**
PC ae C44:4	0.256 ± 0.045	0.269 ± 0.067	0.276 ± 0.071	0.287 ± 0.073	0.1520	0.0748	**0.0059**
PC ae C44:5	1.080 ± 0.256	1.156 ± 0.376	1.198 ± 0.370	1.261 ± 0.406	0.1316	**0.0438**	**0.0036**
PC ae C44:6	1.249 ± 0.318	1.294 ± 0.399	1.395 ± 0.466	1.442 ± 0.452	0.4642	**0.0462**	**0.0070**
SM (OH) C22:1	50.919 ± 10.710	48.931 ± 10.464	48.973 ± 9.044	44.871 ± 10.006	0.2545	0.2829	**0.0016**
SM (OH) C22:2	52.786 ± 11.281	52.478 ± 12.908	52.041 ± 9.473	48.689 ± 11.249	0.8800	0.6952	**0.0473**
SM (OH) C24:1	2.436 ± 0.493	2.360 ± 0.559	2.335 ± 0.383	2.236 ± 0.515	0.3915	0.2109	**0.0303**
SM C18:0	47.830 ± 8.648	48.370 ± 10.166	45.941 ± 8.457	44.168 ± 9.480	0.7347	0.2292	**0.0285**
SM C18:1	22.325 ± 4.680	22.856 ± 5.112	21.305 ± 4.574	20.317 ± 5.263	0.5171	0.2301	**0.0288**
SM C24:0	52.737 ± 11.165	51.142 ± 10.822	51.449 ± 9.778	47.592 ± 10.665	0.3780	0.5020	**0.0105**
SM C26:0	0.528 ± 0.128	0.500 ± 0.153	0.497 ± 0.107	0.476 ± 0.132	0.2441	0.1586	**0.0295**
SM C26:1	1.045 ± 0.385	0.997 ± 0.424	0.944 ± 0.285	0.903 ± 0.278	0.4835	0.1109	**0.0238**

SDMA: symmetric dimethylarginine; lysoPC: lysophosphatidylcholine; PC: phosphatidylcholine; SM: sphingomyelin

### Metabolite patterns by low physical function and sarcopenia

For targeted metabolomics analysis, 289 plasma samples including 57 Con, 104 LPF, 63 S, and 65 SS were subjected to mass spectrometry based targeted metabolites analysis and then datasets were analyzed. Comparisons between the control group and other groups (i.e. LPF, S, and SS) were carried out using an independent student’s t-test. The metabolites changed in different groups when compared with control group (*P* value < 0.05) as shown in Table 3.

**Table 3:** Multivariable analyses on the associations of metabolites with sarcopenia

	LPF vs. Con		S vs. Con		SS vs. Con	
	Estimated OR (95% CI)	*P* value	Estimated OR (95% CI)	*P* value	Estimated OR (95% CI)	*P* value
Age, yrs	1.134 (1.064 ~ 1.209)	**< 0.001**	1.177 (1.082 ~ 1.279)	**< 0.001**	1.266 (1.124 ~ 1.425)	**< 0.001**
Sex	0.360 (0.123 ~ 1.055)	0.063	1.774(0.456 ~ 6.909)	0.408	3.094 (0.439 ~ 21.812)	0.257
BMI, kg/m^2^	1.254 (1.069 ~ 1.470)	**0.005**	0.762 (0.628 ~ 0.923)	**0.006**	0.746 (0.569 ~ 0.979)	**0.035**
BUN, mg/dL	1.042 (0.941 ~ 1.155)	0.482	0.985 (0.880 ~ 1.103)	0.799	1.290 (1.046 ~ 1.591)	**0.017**
KYN,µM	1.275 (0.578 ~ 2.813)	0.548	2.198 (0.953 ~ 5.070)	0.065	3.563 (1.105 ~ 11.481)	**0.033**
C4/Cr, x10^3^	1.830 (1.109 ~ 3.021)	**0.018**	1.956 (1.012 ~ 3.781)	**0.0229**	5.104 (1.461 ~ 17.834)	**0.011**
Hypertension	2.064 (0.840 ~ 5.069)	0.114	0.958 (0.314 ~ 2.922)	0.940	2.827(0.630 ~ 12.686)	0.175
Diabetes mellitus	0.954 (0.284 ~ 3.209)	0.939	2.724 (0.666 ~ 11.144)	0.163	1.577 (0.233 ~ 10.658)	0.641
Hyperlipidemia	1.167 (0.447 ~ 3.047)	0.753	3.162 (0.893 ~ 11.191)	0.074	2.188 (0.387 ~ 11.582)	0.387
CAD	0.461 (0.77 ~ 2.760)	0.397	0.578 (0.075 ~ 4.436)	0.598	0.270 (0.021 ~ 3.538)	0.319
Stroke	1.284 (0.169 ~ 9.763)	0.809	0.363 (0.020 ~ 6.456)	0.490	15.120 (0.365 ~ 626.646)	0.153
CKD	3.449 (0.304 ~ 39.075)	0.317	11.589 (0.902 ~ 148.905)	0.060	3.042 (0.076 ~ 121.061)	0.554
COPD	1.272 (0.381 ~ 4.249)	0.696	2.502 (0.571 ~ 10.967)	0.224	2.270 (0.334 ~ 15.433)	0.402
Osteoprosis	1.779 (0.625 ~ 5.061)	0.280	1.573 (0.439 ~ 5.643)	0.487	6.432 (1.143 ~ 36.200)	**0.035**

C0/Cr: carnitine/creatinine; C4/Cr: C4-carnitine/creatinine; Con: control; CI, confidence interval; KYN: kynurenine; LPF: low physical function; OR, odds ratio; S: sarcopenia; SS: severe sarcopenia

Adjusted factors including sex, age and comorbidities (diabetes mellitus, chronic kidney disease (CKD), hypertension, hyperlipidemia, coronary artery disease (CAD), stroke, chronic obstructive pulmonary disease (COPD), and osteoporosis, BMI, BUNKYN, and C0/Cr or C4/Cr

To test whether metabolites could discriminate participants with LPF, or S from Con, markers were investigated by comparing metabolites in the LPF, S, and SS groups with the Con group and the results were shown by Venn diagram (Fig. 1D) and heatmap (Fig. 1E). A total of 7, 26, and 59 differential metabolites were identified in these groups when compared with the Con group with *P* value < 0.05, respectively (Table 3; Fig. 1). The heatmap revealed high variability of metabolites in each group (Fig. 1E). In the Venn diagram, the overlap in significant metabolites including C0 (F-1), C4 (F-2), C6 (F-3), C18:1-OH (F-4) –carnitine, and KYN (F-5) were presented by box plots in each group.

The changed metabolites in each group (LPF, S, or SS) compared with control group were shown in the summarized scheme (Fig. 1G). These five significant changed metabolites (C0, C4, C6, C18:1-OH-carnitine and KYN) are selected for further multivariable analysis.

### Associations of acylcarnitine with low physical function and sarcopenia

Compared with control group, the relationship between each group and acylcarnitines (C0, C4, C6, or C18:1-OH) which normalized by creatinine (Cr) with significant change was adjusted according to age, sex, diabetes mellitus, CKD, hypertension, hyperlipidemia, CAD, stroke, COPD, osteoporosis, BMI, BUN, and KYN and evaluated by binary logistic analysis. The odds ratios (ORs) are given in Table 2. There are no significant changes in C6/Cr and C18:1-OH (data not shown). After multivariable adjustment, a logistic regression showed that age, BMI, and C4/Cr are more important risk factors to develop LPF and sarcopenia. However, the Estimated OR of BMI was positively associated with LPF but negatively associated with sarcopenia. After multivariable adjustment, it showed that age, BMI, BUN, KYN, and C4/Cr are more important risk factors in severe sarcopenia. The Estimated ORs of C4/Cr levels were 1.830, 1.956, and 5.104 in the LPF, S, and SS groups versus controls, respectively (Table 2). Age, BMI, and C4/Cr identify individuals with sarcopenia from early to severe stages.

**Fig. 2:**
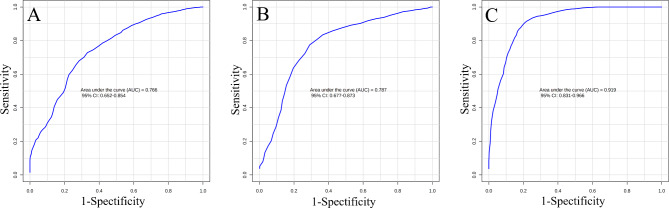
Diagnosis of sarcopenia using candidate metabolite markers. Receiver operating characteristic (ROC) analysis was on a combination of age, BMI, BUN, KYN, and C4/Cr by logistic regression algorithm. ROC curves distinguish participants with low physical function (**A**); participants with sarcopenia (S) (**B**); and participants with severe sarcopenia (SS) (**C**) from controls

The receiver operating characteristic (ROC) curve analysis was performed to evaluate the potential of selected metabolites as biomarkers for sarcopenia or low muscle function diagnosis (Fig. 2). The logistic regression algorithm using a combination of age, BMI, BUN, KYN and C4/Cr demonstrated a better ability to separate SS from Con (AUC: 0.919, Fig. 2C) than S from Con (AUC: 0.787, Fig. 2B) or LPF from Con (AUC: 0.766, Fig. 2A). These results support the potential of combining identified metabolite biomarkers with age, BMI, and BUN to establish an algorithm for monitoring sarcopenia progression.

## Discussion

In the present study, the elevated concentrations of plasma kynurenine (KYN) and acylcarnitines including C0, C4, C6, and C18:1-OH had been identified in older Taiwanese adults with low physical function and sarcopenia. We demonstrated which metabolites are significantly associated with two important sarcopenia traits—muscle mass and function. The concentrations of plasma metabolites such as acylcarnitines, biogenic amines, and phospholipids are associated with muscle mass and physical function and show significant differences among participants with different sarcopenia severities (Fig. 1). The proposed scheme for loss of muscle mass and function indicating the alteration of those metabolites was summarized in Fig. 1. Metabolite patterns could differentiate Con, LPF, S and SS. These metabolic profiling results represent a novel finding and highlight the importance of lipids catabolism in muscle mass and function. Particular metabolites including C0, C4, C6, C18:1-OH and KYN serve as risk factors for the early stages of sarcopenia such as LPF.

The KYN was a significant metabolic indicator associated with muscle mass and strength in this study. Increased KYN metabolism is associated with peroxisome proliferator-activated receptor-gamma coactivator-1α1 expression in skeletal muscle as it indicates the enhancement of energy efficiency and fatigue resistance [40]. Endurance exercise increases skeletal muscle KYN catabolism and plasma kynurenic acid [41]. The KYN pathway is often systemically upregulated when the immune response is activated, which is linked to inflammation [42], and recently, tryptophan metabolism via the KYN pathway has been highlighted as a mechanism of central fatigue [43, 44]. Several studies have revealed chronic inflammation contributes to sarcopenia [45], however, the association with the balance of the KYN pathway is not yet totally understood. In this study, plasma KYN was associated with severe sarcopenia. These findings maybe suggest that KYN is a potential risk factor of severe sarcopenia.

There is mounting evidence that lipids play important roles in the regulation of skeletal muscle mass and function [46]. Carnitine, the carrier moiety of acylcarnitines, plays an essential role in mitochondrial metabolism and muscle bioenergetics. In addition to inborn error [47], dysregulation of acylcarnitine homeostasis and elevated concentrations of plasma acylcarnitines have been linked to a variety of diseases such as higher risk of obesity, insulin resistance, type 2 diabetes [48, 49], and cardiovascular disease [50] and heart failure [51, 52], reflecting dysregulation of fatty acid metabolism in the mitochondria. Increased plasma concentrations of acylcarnitines are markers of incomplete β-oxidation and decreased mitochondrial activity and associate with age [32], low physical performance in elderly males [33], and can be biomarkers of sarcopenia in preoperative gastrointestinal cancer patients [34]. These findings suggest that incomplete β-oxidation might be risk factors for loss of muscle mass or function.

The present study had several strengths. First, we identified the clinical and metabolic signature of sarcopenia. We considered both muscle mass and muscle function, which reflect important pathophysiological aspects of sarcopenia, and identified the specific and shared metabolites among LPF, sarcopenia, the severe sarcopenia, providing important information to further understand the pathogenesis of sarcopenia. Second, after multivariable adjustment, the data indicate that age, BMI and butyrylcarnitine are more important factors to identify individuals with low physical function and sarcopenia. Our findings suggest that acylcarnitines, especially butyrylcarnitine, should be further investigated to elucidate the clinical relevance and potential biomarkers in older adults with sarcopenia. Moreover, the combination of age, BMI, BUN, KYN, and C4/Cr can be important evaluation markers for the early stage of sarcopenia. Previous studies have reported that carnitines are associated with sarcopenia while this study identified acylcarnitines as a predictor of sarcopenia progression.

However, we acknowledged some limitations which must be considered when explaining the study findings. First, we could not conclude the causal relationships between the identified metabolites and muscle status because of the cross-sectional study design. Longitudinal follow-up studies are necessary for the future to clarify the changes in these metabolites related to people on the way to sarcopenia. Second, the relatively higher proportion of LPF participants who are female may conceal the alterations of metabolites in those at an advanced stage. Moreover, our study participants are enrolled from a high-income community which limits the generalizability. Third, we cannot exclude the possibility that there may have been some unmeasured, confounding factors, such as eating habits and lifestyle, after adjusting for the available covariates. Whether chronic systemic disease (heart, liver, or renal dysfunction), or dietary intake over time leads to the change of blood concentration of acylcarnitines needs to be further investigated.

## Conclusions

This metabolomic study raises the importance of acylcarnitines on muscle mass and function. After multivariable adjustment, a logistic regression showed that age, BMI, and C4/Cr are more important risk factors to develop LPF and sarcopenia. And, age, BMI, BUN, KYN, and C4/Cr are more important risk factors in severe sarcopenia. It suggests that age, BMI, BUN, KYN, and C4/Cr can be important evaluation markers for possible sarcopenia.

### Electronic supplementary material

Below is the link to the electronic supplementary material.

**Supplementary Material 1**: **Figure 1**. Study flow diagram. This figure shows the number of participants for metabolites analysis. A total of 491 participants enrolled in this study of which 289 subjects were eligible to participate. Then, targeted metabolomics analysis was performed for 289 samples with completed measurement data and divided into four groups. The groups’ definition was according to Asian countries that used Asian Working Group for Sarcopenia (AWGS) criteria: control (Con, participants who had normal hand grip strength: men ≥ 28 kg, women ≥ 18 kg, and normal gait speed ≥ 1.0 m/s, n = 57), low physical function (LPF, possible sarcopenia with normal muscle mass: men ≥ 7.0 kg/m^2^, women ≥ 5.4 kg/m^2^, n = 104), sarcopenia (S, n = 63), and severe sarcopenia (SS, low HGS and GS, n = 65). The possible sarcopenia (n = 232) was defined by low HGS or GS

## Data Availability

The raw data could be available by connecting the corresponding author.

## List of abbreviations

AWGS: Asian Working Group for Sarcopenia
AUC: area under curve
BMI: body mass index
BUN: blood urea nitrogen
C4: butyrylcarnitine
CAD: coronary artery disease
CI: 95% confidence interval
CKD: chronic kidney disease
COPD: chronic obstructive pulmonary disease
Cr: creatinine
GS: gait speed
HGS: hand grip strength
KYN: kynurenine
LPF: low physical function
ORs: Odds ratios
ROC: receiver operating characteristic
SVM: support vector machine
