# Supplementary material for: Plasma acylcarnitine in elderly Taiwanese: as biomarkers of possible sarcopenia and sarcopenia
Source: BMC Geriatr. 2023 Nov 22;23:769. doi: 10.1186/s12877-023-04485-x (PMC10666394; doi:10.1186/s12877-023-04485-x)

**Plasma Acylcarnitine in Elderly Taiwanese: as Biomarkers of Possible Sarcopenia and Sarcopenia**

**Supporting information**

Chi-Jen Lo^1*^, Chih-Ming Lin^2,3*^, Chun-Ming Fan^1^, Hsiang-Yu Tang^1^, Han-Fang Liu^1^, Hung-Yao Ho^1,4,5^, Mei-Ling Cheng^1,4,6#^

^1^ Metabolomics Core Laboratory, Healthy Aging Research Center, Chang Gung University, Taoyuan City 33302, Taiwan

^2^ Division of Internal Medicine, Chang Gung Memorial Hospital, Taipei 105, Taiwan

^3^ Department of Health Management, Chang Gung Health and Culture Village, Taoyuan City 333, Taiwan

^4^Clinical Metabolomics Core Laboratory, Chang Gung Memorial Hospital, Taoyuan City 33302, Taiwan

^5^Graduate Institute of Biomedical Sciences, College of Medicine, Chang Gung University, Taoyuan City 33302, Taiwan

^6^Department of Biomedical Sciences, College of Medicine, Chang Gung University, Taoyuan City 33302, Taiwan

*These authors contributed equally to this work.

^#^Correspondence: M.-L. Cheng, Metabolomics Core Laboratory, Healthy Aging Research Center, Chang Gung University, Taoyuan City 33302, Taiwan. E-mail: [chengm@mail.cgu.edu.tw](mailto:chengm@mail.cgu.edu.tw); Tel.: +886-3-2118800 (ext.3811); Fax: 886-3-2118700.

**Figure legends**

**Supplementary figure 1. Study flow diagram. This figure shows the number of participants for metabolites analysis.** A total of 491 participants enrolled in this study of which 289 subjects were eligible to participate. Then, targeted metabolomics analysis was performed for 289 samples with completed measurement data and divided into four groups. The groups’ definition was according to Asian countries that used Asian Working Group for Sarcopenia (AWGS) criteria: control (Con, participants who had normal hand grip strength: men ≥ 28 kg, women ≥ 18 kg, and normal gait speed ≥ 1.0 m/s, n = 57) , low physical function (LPF, possible sarcopenia with normal muscle mass: men ≥ 7.0 kg/m^2^, women ≥ 5.4 kg/m^2^, n = 104), sarcopenia (S, n = 63), and severe sarcopenia (SS, low HGS and GS, n = 65). The possible sarcopenia (n = 232) was defined by low HGS or GS.


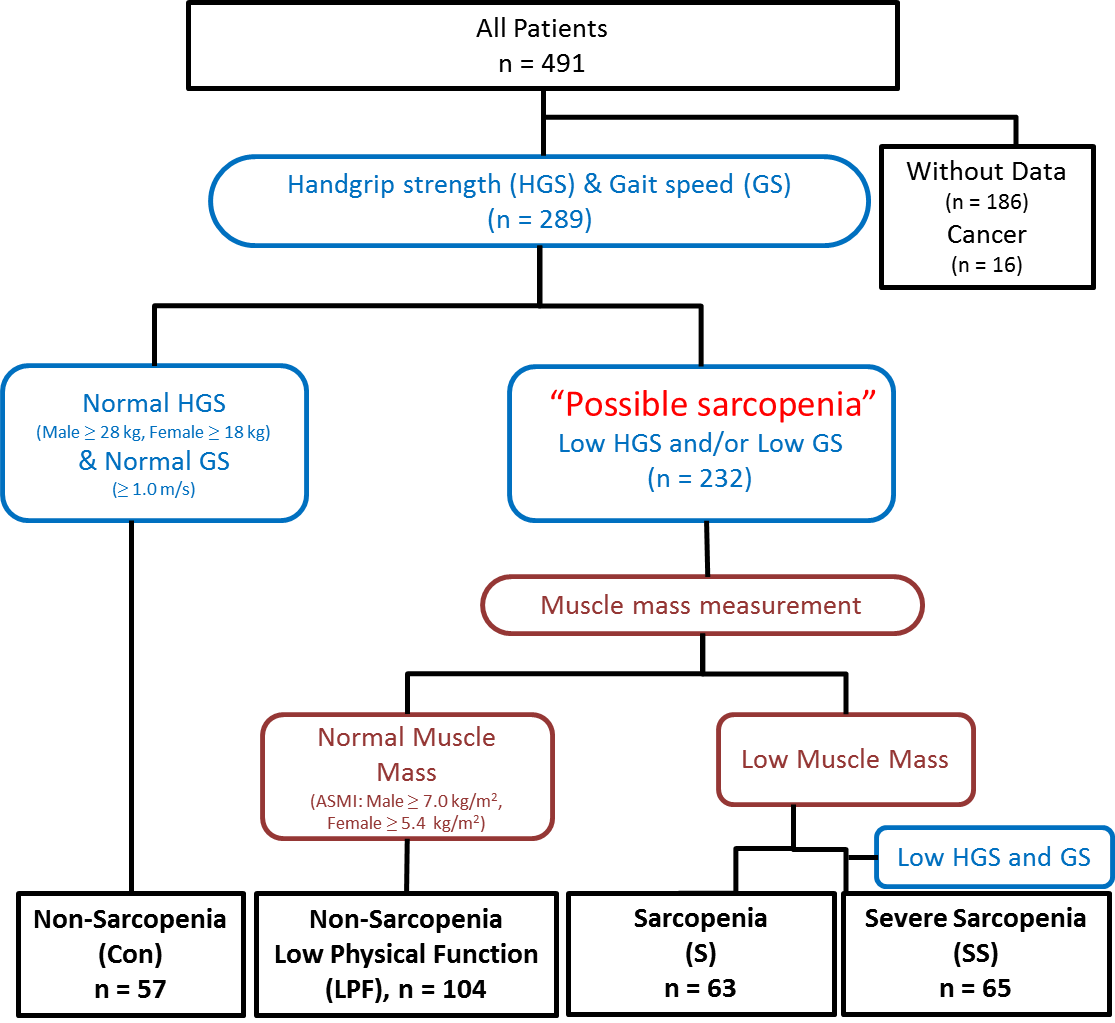

Supplement: Supplementary file 1 — Supplementary Material 1: Figure 1. Study flow diagram. This figure shows the number of participants for metabolites analysis. A total of 491 participants enrolled in this study of which 289 subjects were eligible to participate. Then, targeted metabolomics analysis was performed for 289 samples with completed measurement data and divided into four groups. The groups’ definition was according to Asian countries that used Asian Working Group for Sarcopenia (AWGS) criteria: control (Con, participants who had normal hand grip strength: men ≥ 28 kg, women ≥ 18 kg, and normal gait speed ≥ 1.0 m/s, n = 57), low physical function (LPF, possible sarcopenia with normal muscle mass: men ≥ 7.0 kg/m2, women ≥ 5.4 kg/m2, n = 104), sarcopenia (S, n = 63), and severe sarcopenia (SS, low HGS and GS, n = 65). The possible sarcopenia (n = 232) was defined by low HGS or GS [file 12877_2023_4485_MOESM1_ESM.docx]
